# Characteristics and outcomes of preterm infants with early pulmonary hypertension

**DOI:** 10.1038/s41372-025-02295-0

**Published:** 2025-04-11

**Authors:** Rachel Mullaly, Aisling Smith, Claire Murphy, Seán Armstrong, Orla Franklin, Naomi McCallion, Afif EL-Khuffash

**Affiliations:** 1 Department of Neonatology, The Rotunda Hospital, Dublin, Ireland; 2 Department of Paediatrics, Royal College of Surgeons in Ireland, Dublin, Ireland; 3 Department of Paediatric Cardiology, Children’s Health Ireland at Crumlin, Dublin, Ireland

**Keywords:** Outcomes research, Risk factors, Hypertension

## Abstract

**Objective:**

This study investigates incidence, outcomes and echocardiographic characteristics of preterm infants with early pulmonary hypertension (PH) compared to those without.

**Study design:**

A prospective observational study of infants born <29 weeks gestation between July 2021–March 2024. Echocardiograms were performed at 24–48 h and 36 weeks postmenstrual age (PMA). Early PH was defined as bidirectional or right-to-left shunt across the ductus.

**Result:**

Early PH was identified in 20/166 (12%) infants. These infants had higher mortality than controls (55% vs 11%; *P* < 0.01). Initial echocardiogram revealed differences in twist(*°*) (5.1 vs 7.9; *P* = 0.03), torsion(*°/mm*) (0.29 vs 0.41; *P* = 0.04), systolic time(ms) (146 vs 162; *P* < 0.01) and isovolumic relaxation time(ms) (58 vs 46; *P* < 0.01), with several persistent abnormalities at 36 weeks PMA.

**Conclusion:**

Preterm infants with early PH have higher mortality and distinct echocardiographic profiles, with functional alterations persisting to 36 weeks PMA in survivors. Early identification and targeted management may improve outcomes.

## Introduction

Pulmonary hypertension (PH) in preterm infants is linked to increased rates of mortality and morbidity. It is a complex condition often associated with hypoxic respiratory failure and may manifest as various phenotypes which can be differentiated using echocardiography. The interplay between alveolar and vascular immaturity significantly contributes to the pathogenesis of PH in preterm infants. Several prenatal risk factors for the development of PH in preterm infants have been established, such as maternal diabetes, chorioamnionitis, oligohydramnios, preterm premature rupture of membranes (PPROM), intrauterine growth restriction, pulmonary hypoplasia, and postnatal risk factors such as small for gestational age, invasive mechanical ventilation and hyperoxia [1]. However, these risk factors may also be present in infants who do not develop PH, nor are they prerequisites for the condition’s onset. Our study focuses on a specific cohort of infants who had evidence of PH on their initial echocardiogram. This evidence was often subclinical, highlighting the importance of a screening echocardiogram to identify these infants and detect early-stage PH before overt clinical signs emerge.

The prevalence of early PH in preterm infants is variably quoted in the literature. This is in part due to the lack of a consensus definition and universal echocardiography assessment for all infants within the first few postnatal days. Arjaans et al. reported that 57/104 (55%) infants <30 weeks gestation or with a birth weight <1000 g had a diagnosis of early PH. However, only 104/200 (52%) of the overall eligible cohort had an echocardiogram which was of sufficient quality to be analysed to assign a diagnosis of early PH [2]. In contrast, Giesinger et al. reported that 9/182 (5%) infants <27 weeks in a “hemodynamic screening era” received iNO after an echocardiographic diagnosis of “acute PH” in the first week [3].

Closure of the ductus arteriosus in the setting of early PH can lead to deleterious effects for the infant. Raised pulmonary vascular resistance (PVR) leads to a decrease in preload to the left atrium via the pulmonary veins. The PDA can act as a “pop-off” valve to supply the systemic circulation with adequate cardiac output when there is suboptimal left atrial filling. Therefore, closure of the ductus in the setting of PH can lead to a significant decrease in cardiac output in an already compromised neonate [4]. It is extremely important to obtain an echocardiogram prior to the treatment of a PDA with medical therapy to ensure that there is no evidence of PH. In this study our aim was to assess the incidence of early PH in preterm infants, evaluate clinical outcomes and analyse initial and final echocardiographic parameters in this cohort versus controls. We hypothesised that preterm infants with early PH have higher mortality rates and exhibit distinct echocardiographic parameters, both initially and at 36 weeks postmenstrual age (PMA), compared to preterm infants without early PH. Additionally, we anticipate that differences in cardiac function will persist beyond the acute phase of early PH, indicating a prolonged impact on myocardial function in this vulnerable population.

## Materials/subjects and methods

### Study design and study population

This was a prospective cohort study performed in the Rotunda Hospital NICU, a level three NICU, Dublin, Ireland. Ethical approval was granted from the Hospital Research Ethics Committee. Infants <29 weeks gestation born between July 2021 and March 2024 were considered eligible for inclusion. The cohort was divided into two groups: preterm infants with early PH and without early PH (control group).

### Exclusion criteria

Neonates were excluded from the study if they had a congenital cardiac abnormality present other than a PDA, patent foramen ovale or an incidental finding of a small ventricular septal defect or if they did not obtain an initial screening echocardiogram due to unavailability of a clinician with targeted neonatal echocardiography (TNE) expertise.

### Definition of early pulmonary hypertension by echocardiography

Infants with any degree of bidirectional, or a right-to-left shunt in the absence of congenital heart disease (>24 h after birth) were categorised as having early PH.

### Collected clinical characteristics

The following data points were collected: antenatal, birth and clinical characteristics including gestational age (weeks), birthweight (grams), 5-min Apgar score, sex, mode of delivery, presence of a multiple pregnancy, first pH after delivery, use of antenatal steroids (either complete or partial dosing), presence of oligohydramnios, cardiopulmonary resuscitation (CPR) or adrenaline requirement at birth, whether delayed cord clamping was carried out (≥30 s), if the infant was <3rd centile at birth, the presence of absent or reversed end-diastolic flow on antenatal Doppler, the presence of chorioamnionitis, use of magnesium sulphate (loading dose and/or maintenance infusion administered prior to delivery), presence of pre-eclampsia, PPROM, whether inhaled nitric oxide (iNO) was administered (at any point during hospital stay) and if so, the age (in days) that it was commenced on.

We collected the following echocardiographic measurements: *PDA parameters*: presence of PDA, PDA diameter (mm), direction of shunt, PDA systolic velocity (m/s), PDA diastolic velocity (m/s), pulmonary artery acceleration time (PAAT) (ms); *pulmonary hypertension parameters*: ratio of PAAT to right ventricular ejection time (PAAT:RVET), left ventricular eccentricity index (LVEI); *event timing*: systolic time (ms), diastolic time (ms), isovolumic relaxation time (IVRT) (ms), isovolumic contraction time (IVCT) (ms); *left ventricular (LV) systolic function*: ejection fraction (%), s’ (cm/s), longitudinal strain (%), longitudinal systolic strain rate (1/s); *LV diastolic function*: e’ (cm/s), a’ (cm/s), e’/a’, E/e’; *right ventricular (RV) systolic function*: tricuspid annular plane in systolic excursion (TAPSE) (mm), s’ (cm/s), longitudinal strain (%), longitudinal systolic strain rate (1/s); *RV diastolic function*: e’ (cm/s), a’ (cm/s); *LV rotational measurements*: twist (°), torsion (°/mm), apical rotation (°), basal rotation (°), twist rate (°/s), untwist rate (°/s).

The following cardio-respiratory parameters were collected during the echocardiogram: heart rate, systolic blood pressure (BP) (mmHg), diastolic BP (mmHg), pH, lactate, invasive ventilation, fraction of inspired oxygen (FiO_2_), mean airway pressure, total fluid volume (mL/kg/day) and inotrope use (at time of echocardiogram). Systolic and diastolic BP were invasive if available and non-invasive via cuff if not available; pH and lactate were from arterial, venous or capillary blood gas as available.

### Clinical outcomes

Our primary clinical outcome was mortality (defined as any cause of death before hospital discharge). Secondary clinical outcomes included chronic lung disease (CLD) (defined as any requirement for supplemental oxygen at 36 weeks PMA), severe intraventricular haemorrhage (IVH) (grade 3 or grade 4 IVH as per Papile et al. [5] on cranial ultrasound scan), periventricular leukomalacia (PVL) at discharge (evidence of PVL on final cranial ultrasound scan), necrotising enterocolitis (NEC) (as per modified Bells’ Staging of at least IIA [6]), pulmonary haemorrhage (blood in the endotracheal tube leading to respiratory compromise), inotropic requirement (requirement for initiation of vasoactive medication to treat a low blood flow state at any stage during hospital stay), culture-positive sepsis (positive blood culture not secondary to likely contaminant at any stage during hospital stay) and retinopathy of prematurity (ROP) requiring treatment.

### Echocardiography assessment

Echocardiograms were performed using the Vivid^TM^ Echocardiography System (GE HealthCare, Milwaukee, USA) with a neonatal cardiology multi-frequency 12 MHz probe. Pre-warmed sterile, single-use ultrasound gel sachets were used for scanning. Non-pharmacological oral sucrose was used when deemed necessary to optimise infant comfort. Echocardiography image acquisition was compliant with recently published guidelines from McNamara et al. (2024) [7]. Standard neonatal echocardiography windows included subcostal, parasternal long-axis, parasternal short-axis, apical, high parasternal, aortic arch, ductal and suprasternal views. A thorough evaluation of myocardial structure and function was carried out for all echocardiograms, including a formal assessment for congenital heart disease during the first echocardiogram. Detailed methodologies for acquiring echocardiographic images and the techniques used for offline analysis have been published in prior studies [8–17].

Initial assessment scan was performed at 24–48 h. A repeat echocardiogram was performed at 36 weeks PMA.

### Statistical analysis

Continuous data were tested for normality using the Shapiro-Wilk test and a histogram representation of the data and were summarised as means (standard deviation) or medians [interquartile range], as appropriate. Categorical data were summarised as counts (percentage (%)). Two group analyses were conducted using the student’s *t* test, the Mann–Whitney U test or the Chi-squared test, as appropriate. Logistic regression analysis was performed to assess the association between death and independent variables, including PH, gestational age, first pH, delayed cord clamping and being small for gestational age ( < 3rd centile), with results presented as odds ratios (OR) and 95% confidence intervals (CI). SPSS version 26 was used to conduct the analyses. Statistical significance was considered to be achieved with a *P* value < 0.05.

## Results

One hundred and eighty-one infants were considered for inclusion. Fifteen infants were excluded because they did not have an initial echocardiogram performed due to lack of TNE availability. Early PH was detected in 20/166 (12%). One hundred and forty-six infants did not have early PH and were considered controls. Infants who had early PH were more unwell in the immediate postnatal period, with a lower 5-min Apgar score and first pH after delivery, less delayed cord clamping and a higher requirement for CPR at birth. They were also more likely to be treated with iNO and commenced this treatment at an earlier stage than control infants (Table 1). This cohort also had less favourable cardiorespiratory parameters at the time of initial echocardiogram, with a higher requirement for invasive ventilation, a higher mean FiO_2_, mean airway pressure, inotrope requirement and a lower systolic blood pressure (Table 2). At the time of initial echocardiogram, 5/20 (25%) infants with early PH were receiving high frequency oscillation ventilation (HFOV) compared to 5/146 (3.4%) infants in the control group (*P* < 0.001).

**Table 1:** Clinical characteristics of infants with early pulmonary hypertension.

	Pulmonary hypertension n = 20	Controls n = 146	*P* value
Gestation (Weeks)	25.7 ± 2.0	26.4 ± 1.6	0.08
Birthweight (Grams)	834 ± 300	898 ± 247	0.29
5-Min Apgar Score	7 [4–8]	8 [6–9]	0.01
Male Sex	9 (45)	78 (53.4)	0.48
Caesarean Section	12 (60)	94 (64.4)	0.70
Multiple Pregnancy	5 (25)	36 (24.6)	0.97
First pH After Delivery	7.24 [7.05–7.28]	7.29 [7.23–7.35]	<0.01
Antenatal Steroids	19 (95)	139 (95.2)	0.89
Oligohydramnios	6 (30)	52 (35.6)	0.62
CPR at Birth	3 (15)	3 (2)	0.02
Adrenaline at Birth	1 (5)	2 (1.4)	0.25
Delayed Cord Clamping	7 (35)	93 (63.7)	0.01
Small for Gestation <3^rd^ centile	2 (10)	1 (0.7)	0.04
Absent or Reversed End Diastolic Flow	3 (15)	16 (11)	0.59
Chorioamnionitis	5 (25)	30 (20.5)	0.65
MgSO_4_	19 (95)	132 (90.4)	0.50
Pre-eclampsia	2 (10)	15 (10.3)	0.97
PPROM	8 (40)	54 (37)	0.79
Nitric Oxide (Any Time)	14 (70)	26 (18)	<0.01
Age (in Days) at Commencement of Nitric Oxide	1 [1–3]	5 [1–13]	0.08
Nitric Oxide (>36 weeks PMA)	1 (5)	0	1.0

Values are presented as means ± standard deviation, median [interquartile range], or count (percentage (%)).

*CPR* cardiopulmonary resuscitation, *MgSO*_4_ magnesium sulphate, *PPROM* preterm prelabour rupture of membranes, *PMA* postmenstrual age.

**Table 2:** Cardiorespiratory parameters at the time of first echocardiogram.

	Pulmonary hypertension n = 20	Controls n = 146	*P* value
Heart Rate	162 ± 17	162 ± 14	0.94
Systolic Blood Pressure (mmHg)	46 ± 8	52 ± 9	0.01
Diastolic Blood Pressure (mmHg)	29 ± 8	31 ± 8	0.25
pH	7.28 [7.25–7.32]	7.30 [7.26–7.34]	0.27
Lactate	2 [1.2–3.6]	1.7 [1.4–2.1]	0.20
Invasive Ventilation	15 (75)	63 (43.2)	<0.01
FiO_2_	30 [24–40]	22 [21–25]	<0.01
Mean Airway Pressure	11 [8–12]	8 [8,9]	<0.01
Total Fluid Volume (mL/kg/day)	130 [120–150]	120 [100–140]	0.03
Inotrope Use	9 (45)	5 (3.4)	<0.01

Values are presented as means ± standard deviation, median [interquartile range], or count (percentage (%)).

### Echocardiography parameters on first echocardiogram

Pulmonary haemodynamic parameters from the initial echocardiogram, presented in Table 3, show that infants in the early PH group had elevated pulmonary pressures, as indicated by the presence of a bidirectional shunt across the PDA and lower systolic and diastolic velocities. Additionally, this group had reduced pulmonary artery acceleration time (PAAT) and a lower PAAT to right ventricular ejection time (RVET) ratio.

**Table 3:** Echocardiographic measurements on initial echocardiogram.

	Pulmonary hypertension n = 20	Controls n = 146	*P* value
**PDA and PH parameters**			
*PDA Present*	20 (100)	128 (88)	0.10
*PDA Diameter (mm)*	2.7 ± 0.8	2.3 ± 0.8	0.05
*Bidirectional Shunt*	20 (100)	0	<0.01
*PDA Systolic Velocity (m/s)*	-0.52 [-0.77–-0.43]	1.54 [1.05–1.98]	<0.01
*PDA Diastolic Velocity (m/s)*	0.47 [0.26–0.66]	0.79 [0.43–1.24]	<0.01
*PAAT (ms)*	24 [22–28]	36 [28–43]	<0.01
*PAAT:RVET*	0.18 [0.16–0.23]	0.23 [0.17–0.28]	0.01
*LVEI*	1.4 [1.3–1.5]	1.3 [1.2–1.5]	0.15
**Event Timing**			
*Systolic Time (ms)*	146 ± 37	162 ± 18	<0.01
*Diastolic Time (ms)*	128 ± 28	125 ± 25	0.64
*Isovolumic Relaxation Time (ms)*	58 ± 21	46 ± 15	<0.01
*Isovolumic Contraction Time*	47 ± 17	41 ± 14	0.07
**LV Systolic Function**			
*Ejection Fraction (%)*	77 ± 14	67 ± 8	0.06
*s*‵ *(cm/s)*	4.0 ± 1.1	3.8 ± 0.9	0.48
*Longitudinal Strain (%)*	17.9 ± 5.9	18.5 ± 4.0	0.56
*Longitudinal Systolic Strain Rate (1/s)*	1.8 ± 0.5	1.8 ± 0.4	0.84
**LV Diastolic Function**			
*e*‵ *(cm/s)*	4.6 ± 1.7	4.6 ± 1.2	0.96
*a*‵ *(cm/s)*	6.2 ± 2.0	6.0 ± 1.8	0.61
*e*‵*/a*‵	0.75 ± 0.22	0.81 ± 0.23	0.29
*E/e*‵	11 ± 4	11 ± 4	0.67
**RV Systolic Function**			
TAPSE (mm)	5.2 ± 1.4	5.7 ± 1.3	0.22
*s*‵ *(cm/s)*	5.0 ± 1.4	5.0 ± 1.4	0.89
*Longitudinal Strain (%)*	20.7 ± 7.1	22.3 ± 5.9	0.28
*Longitudinal Systolic Strain Rate (1/s)*	2.4 ± 0.7	2.4 ± 0.6	0.70
**RV Diastolic Function**			
*e*‵ *(cm/s)*	5.5 ± 1.4	5.2 ± 1.4	0.41
*a*‵ *(cm/s)*	8.9 ± 2.2	9.0 ± 2.4	0.91
**LV Rotational Measurements**			
*Twist (*^*o*^*)*	5.1 [2.9–8.5]	7.9 [4.5–11.5]	0.03
*Torsion (*^*o*^*/mm)*	0.29 [0.17–0.47]	0.41 [0.25–0.62]	0.04
*Apical Rotation (*^*o*^*)*	5.6 [2.5–11.3]	8.8 [5.9–13.1]	0.08
*Basal Rotation (*^*o*^*)*	1.0 [0.1–9.2]	0.9 [-1.6–3.7]	0.26
*Twist Rate (*^*o*^*/s)*	108 [85–163]	124 [88–153]	0.84
*Untwist Rate (*^*o*^*/s)*	-104 [-153–-50]	-99 [-145–-65]	0.78

Values are presented as means ± standard deviation or median [interquartile range].

*PDA* patent ductus arteriosus, *PH* pulmonary hypertension, *PAAT* pulmonary artery acceleration time, *RVET* right ventricular ejection time, *LVEI* left ventricular eccentricity index, *LV* left ventricle, *RV* right ventricle, *TAPSE* tricuspid annular plane systolic excursion

Infants with early PH also demonstrated notable differences in myocardial performance, with shorter systolic time and prolonged isovolumic relaxation time (IVRT) compared to controls. No differences in LV and RV function measured by tissue Doppler imaging (TDI) or strain imaging were observed between the groups. However, left ventricular rotational mechanics were impaired in the PH group, as demonstrated by reduced twist and torsion. For detailed values and comparisons, please refer to Table 3.

### Functional echocardiographic measurements on echocardiogram at 36 weeks corrected gestational age

Pulmonary haemodynamic parameters on the 36 weeks PMA echocardiogram, presented in Table 4, indicate that left ventricular eccentricity index (LVEI) remained elevated in the PH group, reflecting persistent differences in ventricular geometry. Additionally, the PH group continued to exhibit a shorter systolic time, suggesting ongoing challenges in overcoming a higher afterload. Regarding functional measurements, infants with early PH demonstrated further impairments in left ventricular rotational mechanics, as shown by reductions in twist, torsion, basal rotation and twist rate. Please refer to Table 4 for a detailed breakdown of these measurements.

**Table 4:** Functional echocardiographic measurements on echocardiogram at 36 weeks corrected gestational age.

	Pulmonary hypertension n = 9 (survivors only)	Controls n = 130 (survivors only)	p
**Pulmonary Hypertension**			
*PAAT (ms)*	38 [26–43]	43 [36–52]	0.06
*PAAT:RVET*	0.22 [0.16–0.24]	0.23 (0.19–0.28]	0.23
*LVEI*	1.4 [1.3–1.5]	1.2 [1.1–1.4]	<0.01
**Event Timing**			
*Systolic Time (ms)*	164 ± 11	176 ± 16	<0.01
*Diastolic Time (ms)*	146 ± 38	133 ± 30	0.23
*Isovolumic Relaxation Time (ms)*	35 ± 11	36 ± 10	0.65
*Isovolumic Contraction Time*	32 ± 11	34 ± 9	0.63
**LV Systolic Function**			
*Ejection Fraction (%)*	72 ± 11	65 ± 9	0.26
*s*‵ *(cm/s)*	4.9 ± 1.0	4.9 ± 1.0	0.92
*Longitudinal Strain (%)*	20.7 ± 3.2	18.7 ± 2.3	0.06
*Longitudinal Systolic Strain Rate (1/s)*	2.0 ± 1.0	1.6 ± 0.9	0.27
**LV Diastolic Function**			
*e*‵ *(cm/s)*	5.8 ± 3.3	6.7 ± 1.8	0.39
*a*‵ *(cm/s)*	9.1 ± 2.6	9.4 ± 2.8	0.69
*e*‵*/a*‵	0.80 ± 0.25	0.88 ± 0.30	0.59
*E/e*‵	15 ± 5	13 ± 4	0.37
**RV Systolic Function**			
TAPSE (mm)	9.6 ± 2.0	9.9 ± 1.3	0.49
*s* ‵ *(cm/s)*	7.6 ± 1.2	7.3 ± 1.3	0.56
*Longitudinal Strain (%)*	23.8 ± 6.8	22.9 ± 7.8	0.80
*Longitudinal Systolic Strain Rate (1/s)*	2.1 ± 0.7	2.6 ± 0.8	0.19
**RV Diastolic Function**			
*e*‵ (cm/s)	10.2 ± 2.3	9.6 ± 1.8	0.57
*a*‵ (cm/s)	14.8 ± 4.0	14.1 ± 3.5	0.55
**LV Rotational Measurements**			
*Twist (*^*o*^*)*	7.1 [4.3–10.7]	14.2 [7.7–19.1]	<0.01
*Torsion (*^*o*^*/mm)*	0.28 [0.13–0.41]	0.47 [0.28–0.67]	0.02
*Apical Rotation (*^*o*^*)*	9.3 [5.8–14.1]	8.1 [3.3–13.7]	0.63
*Basal Rotation (*^*o*^*)*	3.8 [-0.7–6.4]	-5.2 [-9.4–-1.6]	<0.01
*Twist Rate (*^*o*^*/s)*	123 [114–162]	204 [151–258]	0.04
*Untwist Rate (*^*o*^*/s)*	-150 [-200–-105]	-178 [-261–-108]	0.36

Values are presented as means ± standard deviation or median [interquartile range].

*PAAT* pulmonary artery acceleration time, *RVET* right ventricular ejection time, *LVEI* left ventricular eccentricity index, *TAPSE* tricuspid annular plane systolic excursion.

### Clinical outcomes and logistic regression analysis in infants with early pulmonary hypertension

Infants with early PH had a higher mortality rate than controls (11/20 (55%) vs 16/146 (11%) (*P* < 0.01). Infants with early PH also had a higher rate of severe morbidity, with an incidence of severe IVH of 35% (7/20) vs 10.3% (15/146) (*P* < 0.01). See Table 5 for a summary of clinical outcomes in infants with and without early PH. Table 6 summarises logistic regression analysis of the association between various independent variables and the risk of death in infants with early PH. Infants with early PH had an 8.8-fold increased risk of death compared to controls (OR 8.8, 95% CI 2.2–35.3) (*P* < 0.01). Gestational age was a protective factor against mortality (OR 0.4, 95% CI 0.3-0.6) (*P* < 0.01).

**Table 5:** Clinical outcomes of infants with early pulmonary hypertension.

	Pulmonary hypertension n = 20	Control n = 146	*P* value
**Death Before Discharge**	11 (55)	16 (11)	<0.01
**Chronic Lung Disease▪**	5 (56)	73 (56.2)	>0.99
**Severe IVH (Grade 3&4)**	7 (35)	15 (11.5)	<0.01
**PVL**	5 (25)	16 (11)	0.08
**NEC**	2 (10)	23 (15.8)	0.50
**Pulmonary Haemorrhage**	2 (10)	11 (7.5)	0.66
**Inotropes (at Any Stage)**	15 (75)	36 (24.6)	<0.01
**Culture Positive Sepsis**	4 (20)	27 (18.5)	0.87
**ROP Needing Treatment▪**	6 (30)	24 (16.4)	0.14

Values are presented as count (percentage (%)).

▪ denotes outcomes in survivors only

*IVH* intraventricular haemorrhage, *PVL* periventricular leukomalacia, *NEC* necrotising enterocolitis, *ROP* retinopathy of prematurity.

**Table 6:** Association with death in infants with early pulmonary hypertension.

Independent variable	OR (95% CI)	*P* value
Pulmonary Hypertension	8.8 (2.2–35.3)	<0.01
Gestational Age	0.4 (0.3–0.6)	<0.01
First pH	0.8 (0.0–108.0)	0.95
Delayed Cord Clamping	0.8 (0.3–2.4)	0.68
Small for Gestation Age ( < 3^rd^ Centile)	4.3 (0.2–111.7)	0.38

Values are presented as odds ratio (OR) (95% confidence interval (CI)).

*OR* odds ratio, *CI* confidence interval.

## Discussion

In this study we found that preterm infants with early PH had significantly higher mortality and demonstrated distinctive echocardiographic profiles compared to those without early PH. These infants presented with elevated pulmonary pressures, evidenced by altered pulmonary haemodynamic parameters on initial echocardiogram, which were associated with increased respiratory and inotropic support needs. Additionally, early PH was linked to persistent cardiac dysfunction, with notable impairments in left ventricular rotational mechanics that persisted to 36 weeks PMA. These results highlight the significant impact of early PH on both immediate and longer-term cardiac function in this high-risk population.

Regarding risk factors for early PH, our cohort showed no significant differences between groups with regards to the prevalence of PPROM, oligohydramnios or chorioamnionitis. However, a greater proportion of infants in the PH group had a birth weight below the 3rd centile. This aligns with findings from a meta-analysis by Kim et al. which identified an association between oligohydramnios and early PH but found no link with PPROM or chorioamnionitis. Their analysis also confirmed the relationship between early PH and being small for gestational age [18].

Although there were significantly more infants in the early PH group that received iNO (14/20 (70%)), a proportion of infants in the control group also received this therapy (26/146 (18%)). However, initiation of iNO tended to occur later in the control group than in the early PH group (median start of day 5, compared to day 1). The use of iNO in the control group is likely attributable to its off-label use by attending clinicians for hypoxic respiratory failure as opposed to administration following echocardiographic evidence of early PH.

The findings of poorer cardiorespiratory characteristics in the early PH group suggest that preterm infants with early PH have more significant cardiovascular compromise and thus are more haemodynamically unstable from birth. These findings reflect the cardiorespiratory instability associated with PH and the higher level of support that infants with PH require to maintain haemodynamic stability [2]. A suboptimal transition from in utero to ex utero life is associated with delayed transition and we can infer that this leads to a subsequent higher requirement for more intensive resuscitation and intensive care support immediately after birth [19]. Mirza et al. reported that 55% of extremely preterm infants experienced delayed postnatal cardiopulmonary adaptation, which was associated with an increased risk of death or bronchopulmonary dysplasia [20]. While differences in diagnostic criteria between their definition of “delayed postnatal cardiopulmonary adaptation” and our definition of “early pulmonary hypertension” preclude direct comparison, both findings highlight a subset of preterm infants who experience significant cardiovascular compromise early in life and go on to have suboptimal outcomes.

No international consensus exists on the echocardiographic definition of PH in preterm neonates. The World Symposium on Pulmonary Hypertension (Nice, 2018) defines PH as mean pulmonary arterial pressure >20 mmHg in children >3 months of age at sea level [21]. This definition clearly excludes neonates. In our cohort, we diagnosed early PH in any infant who could not, at the time of initial echocardiographic assessment, be treated with medical therapy for a PDA because of bidirectional shunt across the PDA on echocardiogram (regardless of the EL-Khuffash PDA Severity Score). There was no infant in our cohort who had a diagnosis of early PH in the absence of a PDA. Infants with PH had negative PDA systolic velocities. This suggests that the pulmonary pressures were higher than the systemic pressures during systole. PDA diastolic pressures in the PH cohort were also significantly lower than in the control group. The significantly reduced PAAT (24 vs 36 ms) and lower PAAT:RVET ratio (0.18 vs 0.23) in the PH group also indicate raised PVR. A PAAT < 40 ms and a PAAT:RVET ratio of <0.25 are markers of high pulmonary pressures [22, 23]. Interestingly, the median PAAT and PAAT:RVET ratio were <40 ms and <0.25, respectively, in both groups. LVEI is a measurement used as a marker for PH. LVEI reference ranges in preterm infants remain understudied. A cohort study reported reference ranges for LVEI in healthy infants of 1.21 (0.92 – 1.45) [24]. Another study reported the presence of greater than half-systemic pressures with an LVEI of ≥1.3 [25]. We did not see any significant difference in LVEI between groups in our study. In fact, both groups had an LVEI ≥ 1.3. We hypothesise that all preterm infants have a degree of raised pulmonary pressure during the initial few days of age and thus standard measurements for the diagnosis of PH may not be transferrable to extremely preterm infants in the early transitional period.

The significantly shorter systolic time, longer IVRT and the trend towards longer IVCT in the PH group on initial echocardiographic assessment suggests that there is altered cardiac function secondary to PH. Increased afterload secondary to raised PVR explains the impairment of both systolic and diastolic function and is a known haemodynamic phenomenon in PH [15]. Although LV and RV systolic and diastolic function, as measured by ejection fraction, TDI and global longitudinal strain (GLS) did not differ significantly between groups, the reduced twist and torsion apparent in the PH group still suggest subtle impairments in myocardial function. A previous study has reported impaired biventricular function in 30 term neonates with persistent pulmonary hypertension of the newborn (PPHN) when compared to 50 healthy controls. Amongst other measurements, differences were detected in TDI measurements of the RV free wall and GLS [23]. The lack of similar differences between measurements in our study of preterm infants may reflect the inherent myocardial dysfunction known in be present in this cohort in the absence of significantly raised PVR [26]. It has been shown that preterm infants <29 weeks gestation exhibit increasing values for twist, torsion, left ventricular twist rate and left ventricular untwist rate over the first week of life, likely due to a compensatory mechanism to overcome reduced LV compliance [27]. PH has been shown to be associated with a decrease in LV torsion, but not untwist rate, in a group of 44 adult patients. Additionally, a separate group of 42 adults with moderate to severe PH also had reduced LV torsion [28, 29]. The findings from our cohort of preterm infants with early PH aligns with the hypothesis that increased pulmonary pressures can affect myocardial function, even in the absence of overt systolic or diastolic dysfunction.

The persistent differences in echocardiographic parameters at 36 weeks PMA in surviving infants who had early PH suggests that there is persistent cardiac dysfunction in this population, and even after resolution of the acute phase of early PH, there are long-lasting deleterious effects on myocardial function. This impairment in myocardial function is likely primarily driven by altered basal rotation. Studies in adult patients with specific cardiac conditions have correlated abnormalities in left ventricular rotational mechanics with adverse clinical outcomes [30, 31]. Although there was no difference seen between groups in terms of LVEI on first echocardiogram, infants with early PH had a significantly higher LVEI than controls at 36 weeks PMA (1.4 vs 1.2). This suggests that LVEI is perhaps a more sensitive marker for raised pulmonary pressure in the more mature neonate. The difference between groups indicates the persistence of raised PVR that exists past the early PH period. Early PH has been identified as a risk factor for bronchopulmonary dysplasia-associated late PH in extremely preterm infants [32].

There is a significantly higher mortality and severe morbidity rate in infants with early PH compared to controls. Higher rates of IVH have been previously reported in the literature in preterm neonates in association with PPHN [33]. This may be mediated by fluctuating cerebral blood flow in the context of a compromised cardiovascular state. The greater need for inotropic support in the PH group also confirms the association between PH and haemodynamic instability and the need for more intensive support than controls.

Limitations of this study include its observational design and the disparity in group sizes. A matched case-control study may have strengthened the validity of the study, but this was not possible because of the overall small number of neonates diagnosed with early PH. Collaboration between centres with pooling of patient numbers may make larger studies to further investigate this vulnerable patient cohort possible in the future.

In our cohort, initial echocardiographic screening successfully identified a subgroup of preterm infants who had early PH. The significantly higher mortality rate underlines the fact that the effects of early PH are potentially more pronounced in this cohort, perhaps due to immaturity of the cardiovascular system and the possibility of a higher baseline PVR that exists in extremely preterm neonates. It is evident that preterm infants with early PH have unique cardiorespiratory and echocardiographic parameters and criteria for the diagnosis of PH in this cohort needs to be refined, as the “one size fits all” approach does not apply here. The significant difference in echocardiographic parameters at 36 weeks PMA between groups, particularly in left ventricular rotational mechanics, suggests that subclinical cardiac dysfunction persists in these infants which may have long-term cardiorespiratory implications that remain yet unknown. Further research needs to be carried out in this cohort to determine what implications early PH has on long-term outcomes. It remains to be seen whether treatment of subclinical myocardial dysfunction should be actively pursued in this population.

## Data Availability

The datasets generated during and/or analysed during the current study are available from the corresponding author on reasonable request.
